# Securing oil port logistics: A blockchain framework for efficient and trustworthy trade documents

**DOI:** 10.1371/journal.pone.0309526

**Published:** 2024-10-18

**Authors:** Misbah Liaqat, Abdulwahab Ali Almazroi, Junaid Shuja, Ehzaz Mustafa

**Affiliations:** 1 Department of Information Technology, College of Computing and Information Technology at Khulais, University of Jeddah, Jeddah, Saudi Arabia; 2 Department of Computer and Information Sciences, Universiti Teknologi PETRONAS, Seri Iskandar, Malaysia; 3 Department of Computer Science, COMSATS University Islamabad, Abbottabad Campus, Abbottabad, Pakistan; University of Borås: Hogskolan i Boras, SWEDEN

## Abstract

The oil port logistics involves multiple parties including oil tanker owners, port authorities, customs, oil suppliers, and shipping companies. These parties need to exchange a significant amount of data and documentation related to cargo, such as bills of lading, customs declarations, and cargo manifests. This huge amount of data and documentation provides ample opportunities for data manipulation and corruption. Moreover, physical documentation is slow and prone to errors and manipulation. This data can be securely stored and shared between different parties in a tamper-proof and transparent manner using blockchain. Blockchain is a decentralized technology that employs secure hashing and consensus algorithms that can detect any data modification. Hence, this work proposes a blockchain-enabled immutable, and efficient framework for trade documentation in oil port logistics. The proposed framework provides timely processing of oil trade documents and ensures immutability while increasing trust among the trade entities. In addition, this work implements a private blockchain for the execution of smart contracts, which can ensure that all parties involved in the logistics process comply with pre-agreed rules and regulations. Simulation results validate the effectiveness of the proposed framework in terms of transparency, immutability, network latency, throughput, and resource utilization.

## 1 Introduction

Port logistics plays a crucial role in the global movement of goods, and this significance is particularly evident in the oil industry. In international trade, the efficiency of port logistics is essential for the timely and secure transportation of products. The global oil company operations, which are critical to the modern economy, rely on sophisticated logistical networks to ensure the seamless movement of crude oil and processed products at various stages of the supply chain [1, 2]. Oil port logistics includes resource transportation from inside and outside the company. It also includes other activities such as trading, shipping, ordering, and inventory management. There are three processes within the supply chain classified as downstream, upstream, or midstream. these depend on the specific activities carried out at each stage. In Upstream operations, oil extraction and production from platforms located onshore or offshore are handled by various suppliers. Following the extraction process, midstream operations encompass the transportation and storage of the extracted resources in reservoirs or containers, for the benefit of the relevant stakeholders [3]. Ultimately, further downstream along the pipeline, activities take place, including the refining and distribution of oil and gas to customers in the retail, commercial, and critical infrastructure sectors [4, 5].

Various government agencies, academic organizations, and businesses routinely gather data related to oil port logistics, resulting in disparate data sources [6, 7]. Oil port logistics face numerous challenges when attempting to share this data. Initially, there exists a deficiency in trust across diverse government organizations, academic institutions, and corporations. Furthermore, the security measures in place for safeguarding personal data are insufficient. Furthermore, the level of data traceability is inadequate [8]. The traditional oil data exchange utilizes a centralized method. It requires storing and consolidating data on a single server under the authority of an authorized intermediary. These authorities are responsible for managing the transfer and distribution of the data consistently [9, 10]. However, this traditional sharing model suffers from many security issues. These are malicious modification of centralized data, lack of transparency, loss of control for data owners, a single point of failure, and difficulty in tracing data.

Blockchain ensures transparency, dependability, traceability, and other complex logistics operations securely It is an optimal solution for supply chain systems and also makes information exchange easier. In case of node failures, blockchain provides fault tolerance capabilities due to its distributed ledger technology [11, 12]. In contrast to centralized systems, blockchain’s decentralized nature provides fault-tolerant capabilities to different attacks to prevent important data and the whole system. Moreover, it also eliminates the dependency on intermediaries for transaction authentication [13, 14].

**Fig 1:**
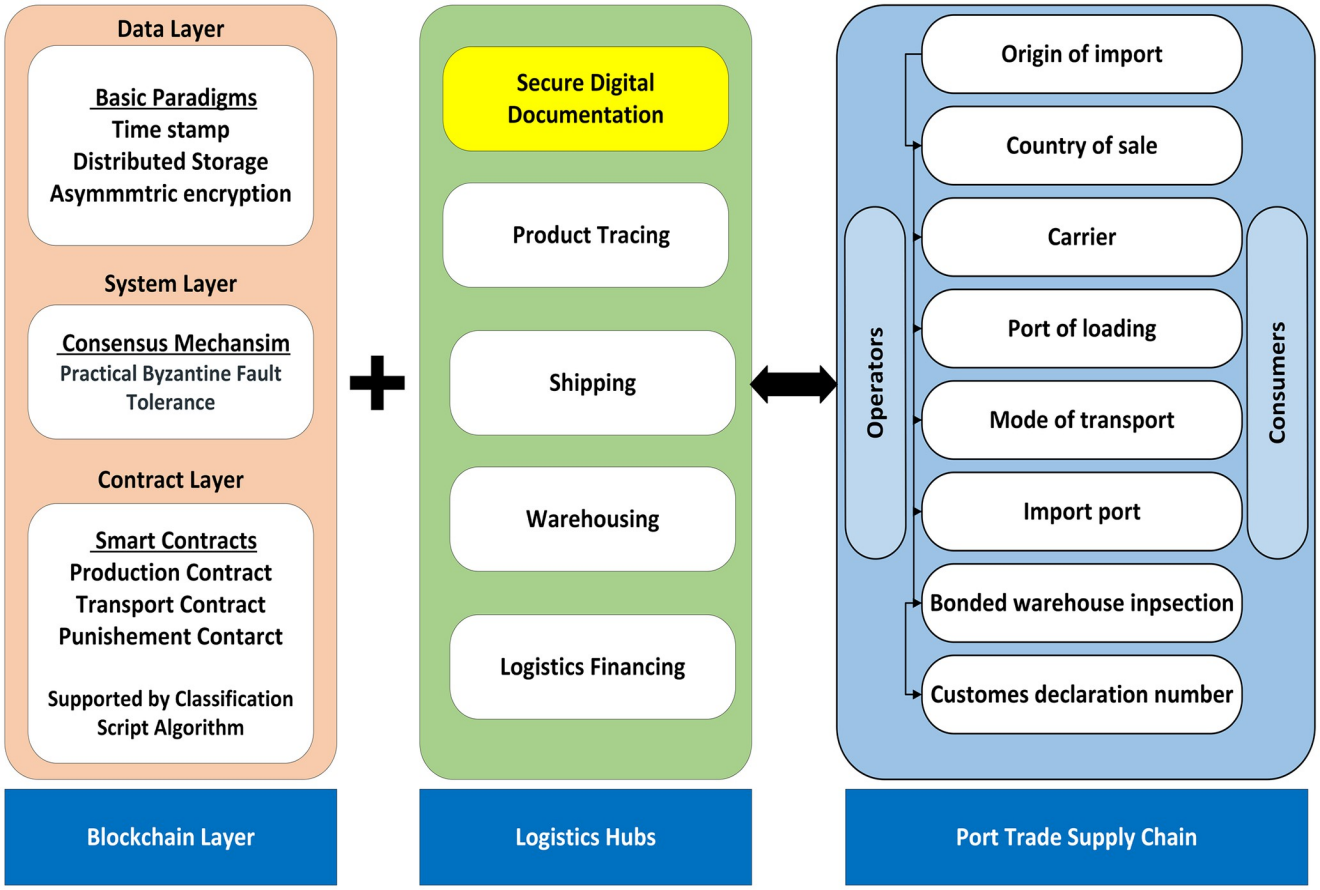
Complete overview of blockchain-based oil port logistics.

Blockchain is an optimal solution to ensure a secure exchange of information in oil port logistics, which is important in these systems. In its peer-to-peer architecture, the nodes are distributed into multiple nodes to ensure the immutability and integrity of data [15, 16]. Blockchain has three categories including private, public, and hybrid blockchains. In public blockchains, all the nodes have equal access rights to read and write the respective data [17]. In a private blockchain, a single organization uses it to allow strict control over node permissions. In contrast to these, hybrid blockchain allows different degrees of access to different groups of nodes to the data. These efficient categories show the significance of blockchain adaptability in different organizations and operations such as oil port logistics. Moreover, it also can deal with different documentation processes such as custom declarations and bills of lading [18, 19] Fig 1.

Motivated by this discussion, this work provides a blockchain-based secure, and trustworthy framework for trade documentation in oil port logistics. The proposed framework ensures data integrity in oil port logistics by using blockchain’s immutability, decentralized consensus, and cryptographic security. By storing trade documents on a private blockchain, any unauthorized data manipulation becomes detectable due to the immutable ledger. Additionally, the use of secure hashing with SHA-256 and consensus algorithms guarantees that only verified transactions are recorded, preventing tampering and ensuring the authenticity of all trade documents. The private blockchain is used because privacy and data confidentiality are paramount in port operations, where sensitive information such as cargo details, shipping schedules, and contractual agreements need to be securely managed among authorized stakeholders. Moreover, the proposed work utilizes Hyperledger Fabric because it offers better scalability, performance, and customized features, which are crucial for the specific needs of port operations. The following are the novel contributions of this research.

- This research aims to address the issue of data manipulation and provide an immutable framework for trade documentation in oil port logistics with its secure hashing and consensus algorithms.
- The private blockchain is used for the management of oil port logistics trade documents. The primary objective is to establish an immutable and efficient blockchain framework.
- The proposed approach uses private blockchain to automate the execution of smart contracts. These smart contracts are strategically designed to enforce compliance with pre-agreed rules and regulations, ensuring a streamlined and trustworthy logistics process.
- We provide an open-source code along with the applications of the proposed framework which is available at: https://github.com/aizazmustafa/Blockchain-based-Oil-port.

The rest of the article is organized as follows: Section 2 provides the related work. Section 3 presents the proposed framework. In section 4, we provide implementation details. Section 5 provides system testing and results. Finally, Section 6 concludes the article.

## 2 Related work

Researchers utilized optimization approaches, data-driven analytics, and machine learning to handle a significant volume of data during the exploration and production in oil port logistics. Despite these, the common use of separate data systems highlights the importance of prioritizing data integration and interoperability. To address this, a standardized machine learning project is proposed, offering data management functionalities through the Open Subsurface Data Universe (OSDU) platform [20]. The upstream markets of the oil and gas sector face challenges such as equipment management, resource monitoring, and preventing the unauthorized disclosure of confidential vendor data [21]. To address the poor asset integrity management and plenty of monitoring devices that lead to human error and costly fines, authors proposed an Industrial Internet of Things (IIoT) architecture that uses blockchain technology for real-time surveillance of oil field activities [22]. This framework integrates IoT device monitoring with the immutable potential of blockchain to increase efficiency of oil field operations. The traceability in blockchain technology is important for maintaining data integrity [23]. The authors in [14] provided the security of IIoT network nodes, gateways, and servers by integrating permissioned blockchains. The proposed approach proved the effectiveness of threshold signature-based systems for authentication. Moreover, authors also proposed reputation-based systems for authorization, and group signature-based systems for revocation.

In the context of the supply chain, data processing, replication, integrity, and security issues impact both midstream and downstream operations [24]. External attacks and the presence of duplicate contracts and transactions among several parties lead to inaccurate and delayed transactions, fraud, mistrust, and increased operational and validation costs. To this end, authors [25] proposed PeTroShare. This platform aimed at streamlining public trading and exchange of reliable, privacy-conscious, and cost-effective crude oil, to improve midstream market functionality. They concluded that PeTroShare may be used for anonymous transportation processes, leveraging blockchain’s privacy capabilities to reduce rates and enhance service quality (Table 1).

**Table 1:** Related work summary with their limitations.

Paper	Year	Application	Methodology	Contributions	Advantages	Limitations
[20]	2023	Data Integration	OSDU platform for data management functionalities	Importance of gathering and translating data into critical business information to enhance exploration and production, refining and manufacturing efficiency	Improve data integration and interoperability	Integration requires significant investments
[14]	2021	Data Sharing and Security	Decentralized and immutable blockchain framework	Improved data security and trust	Securely share large data volumes in the oil and gas industry	Regulatory compliance, integration with legacy systems
[25]	2021	Data Integrity and Security	PeTroShare platform for secure oil trading	Anonymous transportation, reduced costs, improved service quality	Streamline crude oil trading with privacy and cost-effectiveness	Scalability, regulatory compliance
[26]	2021	Data Security and Automation	SmartOil system with smart contracts for data verification	Encryption for data privacy, smart contracts for error reduction	Securely store and automate oil and gas data	Integration with existing systems, adoption challenges
[32]	2024	Safety and Security	Blockchain with IIoT	Enhanced safety, Real-time monitoring	Safe and secure transmission in pipelines	Compatibility with existing Infrastructure, Limitations of IoT devices.
[33]	2021	Shipment nomination	KNPC Blockchain	Improved Communication and Collaboration	Increased Efficiency, Improved security	Technical complexity, scalability
This Paper	2024	Framework for immutable trade documents	Hyperledger framework	Digital trade documentation	Secure, efficient and trustworthy trade documents	Nil

The authors presented a blockchain-powered system named SmartOil designed to securely store a wide range of data generated by several operations, including crude oil extraction and customer transactions [26]. Their deployed encryption ensures data cannot be accessed by unauthorized parties during transit via insecure networks. Moreover, SmartOil utilizes smart contracts to automate the verification of blockchain data, minimizing errors in data mismatch, fraud, and counterparty settlements. The authors in reference [27] suggested a similar use for the downstream market that leverages the inherent SHA-1 capabilities of a permissioned blockchain.

The oil and gas industry involves several stakeholders such as governmental authorities, banks, regulatory bodies, academic institutions, and others. Safe transmission of data between these organizations is essential [28]. The authors employed a permissioned blockchain to construct a business ballot system that integrates digital signatures and identities. Focusing on the oil and gas network, heavily dependent on consortiums, their primary concern is information loss, including personal data, customer information, and partner information [29]. The authors concluded that Data privacy in the oil and gas sector may be safeguarded through private and consortium blockchains. This improves integrity and security via immutability and significantly reduces the likelihood of data leakage by demanding a substantial amount of computing resources to attempt such actions. Furthermore, several cryptographic methods may enhance security, ensuring the confidentiality of copyright information related to oil data.

Despite these efficient schemes, none of the previous works incorporated permissioned blockchain for the immutable oil port logistics trade documents. To fill this gap, we aim to provide an approach that involves the implementation of a private blockchain to automate the execution of smart contracts, thereby allowing strict control over node permissions to ensure the trustworthiness of trade documents.

## 3 Proposed framework

**Fig 2:**
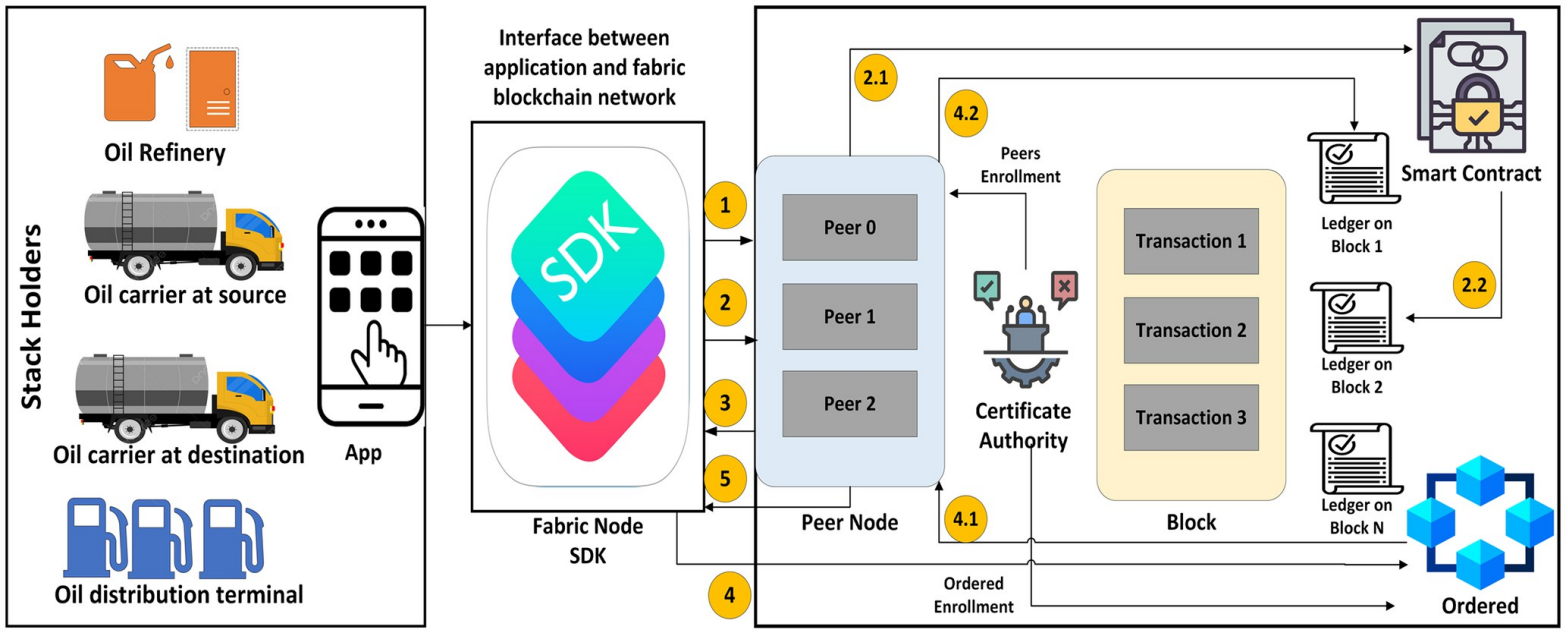
Proposed hyperledger framework for secure digital documentation.

Our proposed framework is depicted in Fig 2. We utilize the Fabric SDK node as an interface connecting the user application to the Fabric blockchain network. The left section presents a list of stakeholders and their respective applications. Here, stakeholders are linked through the application with the Fabric SDK node. The right section illustrates the complete procedure of the Fabric blockchain network, encompassing peer nodes, ordered nodes, blocks, ledgers, smart contracts, and the certificate authority. Initially, the application triggers a transaction towards the Fabric SDK. The Fabric SDK establishes a connection with the peer node in the first step. Subsequently, in the second step, the application invokes a chaincode (proposal), followed by the peer invoking the chaincode with the proposal. The chaincode then generates the queue or updates the proposal response. In the third step, the peer sends a proposal response to the application. In the fourth step, the application requests that the transaction be ordered. The ordered node follows this step and dispatches the transaction to peers in blocks, after which the peer updates the ledger and employs transaction blocks. In the last step, the peer node sends the event of ledger update to the application. In this process, the certificate authority sends peer enrollment to the peer node and ordered enrollment to the ordered node. Additionally, this authority issues certificates to the stakeholders in the left portion.

### 3.1 Transaction flow

**Fig 3:**
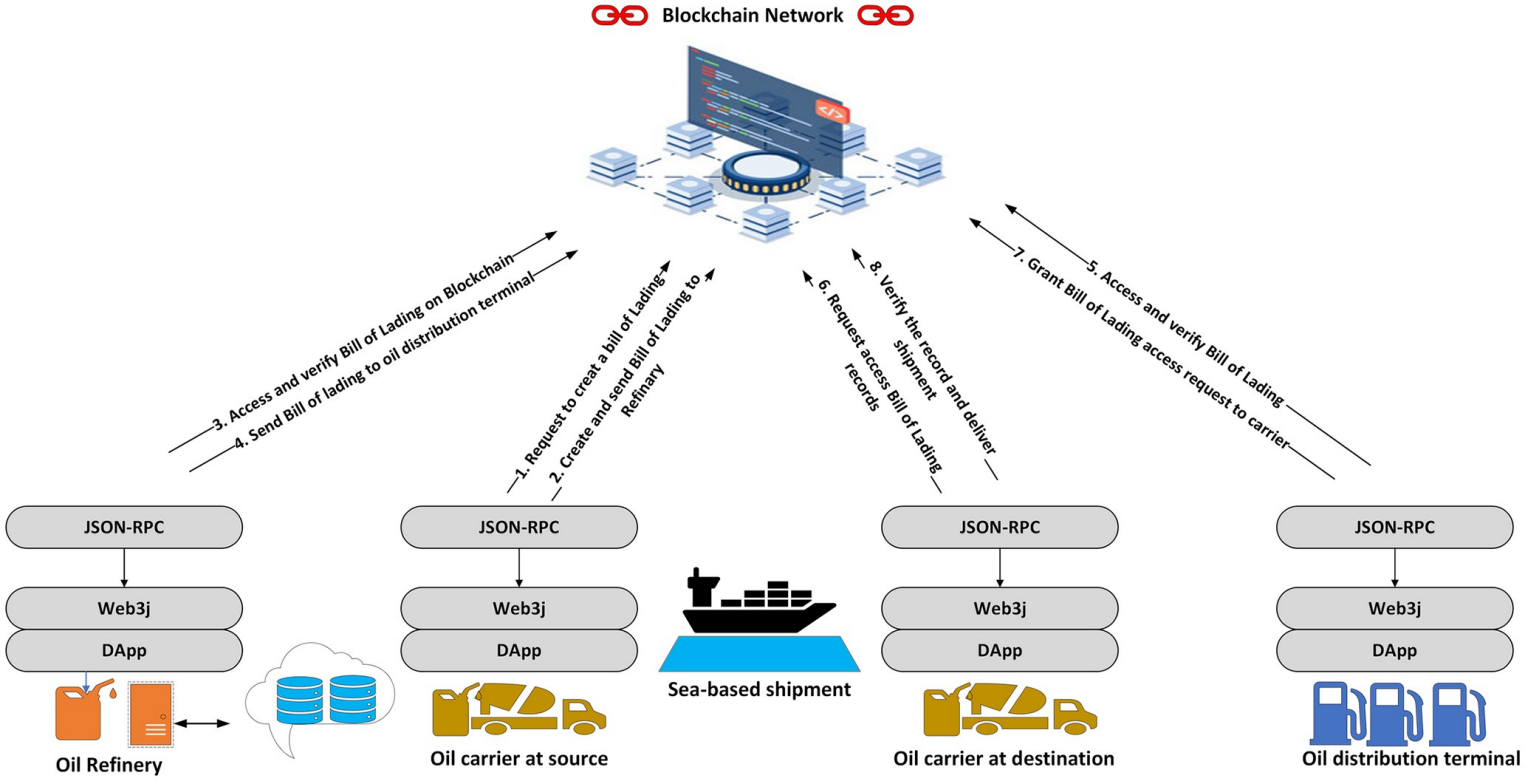
Flow for secure trade documents through smart contracts built on blockchain technology.

This application enables stakeholders to interact with the blockchain network, with the SDK Fabric node serving as the bridge between the application and the network. We aim to provide a secure and efficient framework for managing oil port logistics trade documents using blockchain technology. We take an example scenario of the bill of lading process. In our scenario, first, the oil carrier at the source sends a request on the blockchain network to create a bill of lading for the oil refinery. After this step, the bill of lading is then created and recorded on the blockchain. After receiving the corresponding bill of lading, the oil refinery accesses and verifies it on the blockchain. Here the status of the bill is checked. If the bill is valid, the refinery sends the bill to the oil distribution terminal, which also accesses and verifies it. The oil carrier at the destination requests access to the bill of lading record. Then, the oil distribution terminal grants access. Finally, the carrier at the destination verifies the record on the blockchain and proceeds with delivering the shipment. Throughout this process, the customs department holds the unique authority to update specific customs-related information within the bill of lading. After verifying that the bill has been created and verified on the blockchain, the customs department can make its updates and permanently save the altered version on the ledger. Moreover, the system allows access to specific data within the bill of lading, such as pricing information or sensitive cargo details, only to authorized parties like the shipper, receiver, and customs officials. Fig 3 shows the complete flow of the proposed system.

### 3.2 Layered architecture of proposed system

**Fig 4:**
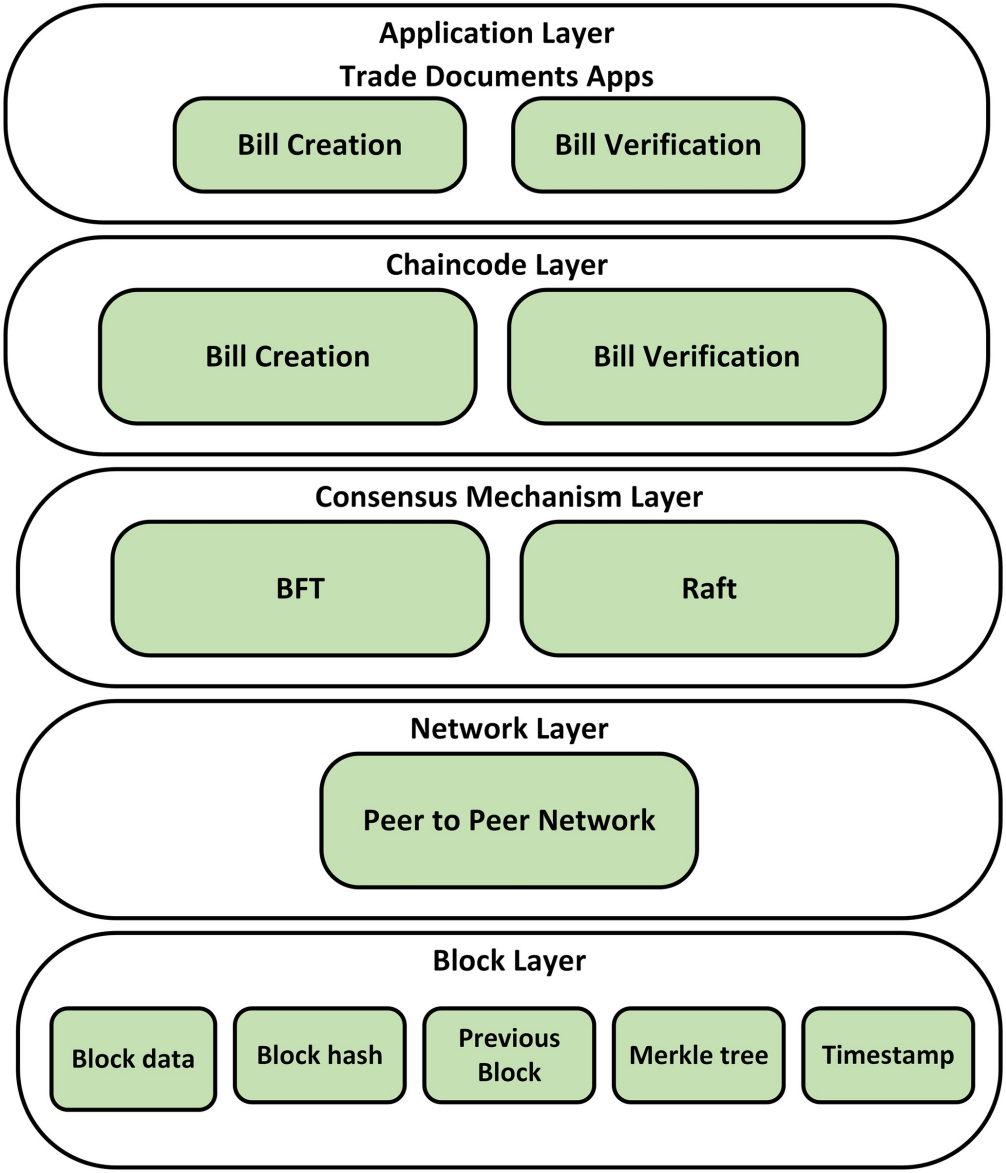
Layered architecture of the proposed framework.

Fig 4 shows the layered architecture of the proposed system consisting of five layers. These layers include the Application Layer, Chaincode Layer, Consensus Mechanism Layer, Network Layer, and Block Layer.

#### 3.2.1 Application layer

At the top of the proposed framework, the application layer acts as the uppermost layer responsible for interacting with users and managing bill-related processes. In our layered architecture, we consider two different applications. the first one is bill creation and the second one is bill verification. The first application which is bill creation enables the oil carrier at source to generate a bill on the blockchain network. On the other hand, bill verification allows other verified entities such as oil refineries, oil distribution terminals, and customs officials to access and verify each bill stored on the blockchain network. This application layer ensures a reduction in fraud and error and also removes the need for manual bill exchange between respective entities by integrating distinct applications.

#### 3.2.2 Chain-code layer

The purpose of the chain-code layer is to provide executable logic from predefined rules and regulations to which the stakeholders agreed. In this layer, we also provide two different chain-codes, the first one is bill creation and the second one is bill verification. As described above, bill creation allows the oil carrier at source to generate a bill of lading and store it on the blockchain. Moreover, additional details could also be added by this entity such as pricing information to the bills. After this step, other stakeholders such as oil refineries, oil carriers at the destination, and oil distribution terminals can verify and update the existing bills on the blockchain. These chain-codes ensure the immutability of these documents over the network.

#### 3.2.3 Consensus mechanism layer

We use consensus mechanisms to ensure the prevention of forks and data integrity, and also to validate the transaction from all the entities. Consensus mechanism also ensures that all the stakeholders agree on a single and consistent state of the ledger. To ensure high security due to trade documents, we consider the Raft and Byzantine Fault Tolerance (BFT). The BFT algorithm is specifically used for ordering transactions. Additionally, BFT ensures that every peer has the same list of transactions on its ledger.

#### 3.2.4 Network layer

This layer in the blockchain network is used to manage how nodes are connected in the network. It includes four main components. These components are channels, ordered nodes, peer nodes, and certificate authority. Channels are private communication lines. Each line is for specific groups of participants to communicate privately. In our blockchain system, different groups like oil refineries, carriers, and distribution terminals have a single line for transactions. Second, the ordered nodes make sure all transactions are in the right order, put them into blocks, and share them with others. They make the process secure, efficient, and reliable. Peer nodes keep each participant on a consistent state of the ledger. They ensure the records are consistent and each node can trust them. They check transactions, execute smart contracts, and agree in consensus on transaction validity. Finally, the Certificate Authority (CA) provides digital certificates to identify the participants on the network. Only people with the right certificates can access and make transactions.

#### 3.2.5 Block layer

The block has details such as a cryptography hash of the block and the preceding block’s hash in the blockchain’s sequence and respective data within the block. These data ensure the security and integrity of blockchain technology and guarantee the immutability and transparency of data throughout the network. The hash of the block is used to protect the content and make it temper-proof. The hash of the previous block provides a link to the previous block to ensure the continuity and reliability of the blockchain’s distributed ledger system [30, 31].

**Algorithm 1** CreateBillOfLading

1: **procedure** createBillOfLading(ctx, billId, shippersDetail, receiverDetail, thirdPartyCharges, productInfo, carrierDetail, transportationMeans, taxDetail)

2:  Check if the transaction submitter has the ‘oilCarrierAtSource’ role

3:  **if** !checkRole(ctx, ‘oilCarrierAtSource’) **then**

4:   Throw error: ‘Only Oil Carrier at Source is allowed to create bills of lading.’

5:  **end if**

6:  Get the current date and time in ISO format

7:  Create a new bill of lading object

8:  Record the creator’s identity: *ctx.clientIdentity.getID()*

9:  Set initial status to ‘created’, and initialize custom options and shipment details

10:  Save the bill of lading in the ledger using *ctx.stub.putState*

11:  Return the created bill of lading

12: **end procedure**

13: **procedure** getBillOfLading(ctx, billId)

14:  Retrieve existing bill from the ledger using *ctx.stub.getState*

15:  **if** existingBillBytes and existingBillBytes.length > 0 **then**

16:   Parse and return the bill of lading object

17:  **else**

18:   Return null

19:  **end if**

20: **end procedure**

21: **procedure** checkRole(ctx, requiredRole)

22:  Get client identity from *ctx.clientIdentity*

23:  Get roles attribute from client identity

24:  Check if *requiredRole* is present in roles

25:  Return true if present, false otherwise

26: **end procedure**

**Algorithm 2** VerifyBillByOilRefinery

1: **procedure** verifyBillByOilRefinery(ctx, billId)

2:  **if** !checkRole(ctx, ‘oilRefinery’) **then**

3:   Throw error: ‘Only Oil Refinery is allowed to verify bills of lading.’

4:  **end if**

5:  existingBill ← Retrieve existing bill of lading using getBillOfLading(ctx, billId)

6:  **if** existingBill.status is not ‘created’ **then**

7:   Throw error: ‘The bill of lading has already been verified.’

8:  **end if**

9:  existingBill.status ← ‘verified’

10:  Save the updated bill of lading in the ledger using ctx.stub.putState(billId, Buffer.from(JSON.stringify(existingBill)))

11:  Return the updated bill of lading in JSON format

12: **end procedure**

**Algorithm 3** DeliverShipmentByOilCarrieratDestination

1: **procedure** deliverShipmentByOilCarrieratDestination(ctx, billId)

2:  **if** !checkRole(ctx, ‘OilCarrieratDestination’) **then**

3:   Throw error: ‘Only Oil Distribution Terminal is allowed to deliver shipments.’

4:  **end if**

5:  existingBill ← Retrieve existing bill of lading using getBillOfLading(ctx, billId)

6:  **if** existingBill.status is not ‘verified’ **then**

7:   Throw error: ‘The bill of lading needs to be verified before delivery.’

8:  **end if**

9:  existingBill.status ← ‘delivered’

10:  Save the updated bill of lading in the ledger using ctx.stub.putState(billId, Buffer.from(JSON.stringify(existingBill)))

11:  Return the updated bill of lading in JSON format

12: **end procedure**

**Algorithm 4** UpdateCustomOptions

1: **procedure** updateCustomOptions(ctx, billId, customOptions)

2:  existingBill ← Retrieve existing bill of lading using getBillOfLading(ctx, billId)

3:  **if** existingBill.status is not ‘created’ and is not ‘verified’ **then**

4:   Throw error: ‘Customs can only update custom options for bills in the “created” or “verified” state.’

5:  **end if**

6:  existingBill.customOptions ← customOptions

7:  Save the updated bill of lading in the ledger using ctx.stub.putState(billId, Buffer.from(JSON.stringify(existingBill)))

8:  Return the updated bill of lading in JSON format

9: **end procedure**

**Algorithm 5** UpdateShipmentDetails

1: **procedure** updateShipmentDetails(ctx, billId, shipmentDetails)

2:  **if** !checkRole(ctx, ‘shippingCompany’) **then**

3:   Throw error: ‘Only Shipping Companies are allowed to update shipment details.’

4:  **end if**

5:  existingBill ← Retrieve existing bill of lading using getBillOfLading(ctx, billId)

6:  **if** existingBill.status is not ‘verified’ and is not ‘delivered’ **then**

7:   Throw error: ‘Shipping companies can only update shipment details for bills in the “verified” or “delivered” state.’

8:  **end if**

9:  existingBill.shipmentDetails ← shipmentDetails

10:  Save the updated bill of lading in the ledger using ctx.stub.putState(billId, Buffer.from(JSON.stringify(existingBill)))

11:  Return the updated bill of lading in JSON format

12: **end procedure**

## 4 Implementation details

As a case study to evaluate the proposed system, we consider the bill of lading and to ensure the immutability and security of trade documents, we use a permissioned blockchain network. The pre-agreed rules and regulations in the smart contracts are designed to ensure compliance and streamline operations among all parties involved in the oil port logistics process. These rules include conditions for document submission, verification timelines, and approvals of workflows. Payments are automatically triggered when all required documents (e.g., bills of lading) are validated and consensus is reached among authorized parties. The proposed system relies on Fabric, a distributed operating system, to establish trust among users. The implementation of Hyperledger Fabric utilizes an append-only replicated ledger data structure and ensures the secure and digital documentation of transaction history. Hyperledger Fabric introduces the execute-order-validate model, bringing in improved scalability and the flexibility of adaptable trust assumptions. This innovative framework effectively addresses challenges such as non-determinism and security issues, including common problems like resource depletion and performance bottlenecks often encountered within blockchain architectures.

We execute five different algorithms to ensure that the complete process is efficient and trustworthy. In Algorithm 1 “CreateBillOfLading”, we define a procedure to generate a bill of lading. The input parameters include the context (ctx), unique bill identifier (billId), shipper’s details, receiver’s details, third-party charges, product information, carrier details, transportation means, and tax details. The procedure first checks if the transaction submitter possesses the ‘OilCarrierAtSource’ role. If not, the function throws an error. Subsequently, it retrieves the current date and time in ISO format, creates a new bill of lading object, records the creator’s identity, sets the initial status to ‘created,’ and initializes custom options and shipment details. The bill of lading is then stored in the ledger using *ctx.stub.putState*, and the created bill of lading is returned as output. Here, “GetBillOfLading” is a procedure to retrieve an existing bill of lading from the ledger based on the provided bill identifier (billId). The algorithm first fetches the existing bill from the ledger using *ctx.stub.getState*. If the retrieved bill is not empty (existingBillBytes.length > 0), it parses and returns the bill of lading object. Otherwise, it returns *null*. Finally, the “checkRole” procedure provides a utility function to verify if a user, identified by the context (ctx), possesses a specific role (requiredRole). The procedure retrieves the client identity from *ctx.clientIdentity*, obtains the roles attribute from the client identity, and checks if the required role is present in the roles. It returns *true* if the role is present and *false* otherwise. This role-checking functionality is crucial in ensuring that only authorized users, specifically, with the “OilCarrierAtSource” role, can execute the “CreateBillOfLading” procedure.

In the “VerifyBillByOilRefinery” algorithm, we execute a procedure to verify a bill of lading by the oil refinery. The input parameters include the context (ctx) and the unique bill identifier (billId). The algorithm begins by checking if the user executing the transaction possesses the ‘oilRefinery’ role using the checkRole utility function. An error occurs if the user lacks the required role, ensuring that only authorized user from the oil refinery can perform bill verification. In getBillOfLading procedure, if the status of the retrieved bill is not ‘created,’ indicating that the bill has already been verified, an error is thrown to prevent duplicate verification. Upon successful verification, the status of the bill is updated to ‘verified,’ and the modified bill of lading is saved back to the ledger using ctx.stub.putState. The updated bill of lading in JSON format, is returned as output. This algorithm ensures that the verification process is executed securely and transparently, with strict role-based access controls to maintain the integrity of the oil logistics system.

In algorithm 3, we provide the procedure for shipment by the oil carrier at the destination. For this purpose, we consider two input parameters including the identifier of the bill of lading (billId) and the context (ctx). In the first step, the role is verified that the respective user performing the transactions has the role of ‘OilCarrieratDestination’. This is because we restrict only the authorized person from the oil carrier at the destination to perform this transaction. In case of unauthorized access, the system returns an error. After verification, the algorithm acquires the current bill of lading using getBillOfLading function. This procedure determines if the acquired bill has the status of verified before the delivery. In case of illegal verification status, the algorithms throw an exception, and in case of successful verification, the status of the bill is changed to delivered ensuring that the shipment is delivered and the procedures have been completed. After these operations, the amended bill of lading is then stored in the ledger using ctx.stub.putState. Finally, the algorithm returns the revised bill of lading in JSON format.

In the algorithm “UpdateCustomOptions”, we provide the process of updating those custom options that are associated with the bill of lading. This algorithm takes three inputs. First, the unique identifier of the bill of lading which is represented as (billId). The second is the context (ctx) and the third one is the new custom options (customOptions). By using the function getBillOfLading, the algorithm first acquires the existing bill of lading and then checks its status to ensure that it is in the status of both ‘created’ and ‘verified’. If these statuses are not verified, the algorithm returns an error that customs can only update those bills that have these specific statuses. Through this restriction, we ensure that custom’s related modifications are made in the appropriate stages of the logistic process. On the other hand, the custom-related details are updated in the respective bill of lading and the modified bill is stored again on the ledger. After these, the algorithm returns the output in terms of the updated bill of lading in JSON format

In Algorithm 5 “UpdateShipmentDetails”, we provide the process for updating shipment details associated with a bill of lading. This algorithm takes three input parameters. The first one is the context (ctx), the second one is the unique identifier of the bill of lading (billId), and the third one is new shipment details (shipmentDetails). After taking these inputs, the algorithm starts by checking if the transaction submitter has the ‘shippingCompany’ role using the checkRole utility function. If not, an error is thrown, indicating that only shipping companies are allowed to update shipment details. After this verification, the existing bill of lading is acquired using the function getBillOfLading. Then the algorithm verifies the status of the bill to ensure that the bill is in the status both ‘verified’ or ‘delivered’. If this verification is successful, the algorithm updates the shipment details accordingly, and the updated bill is stored on the ledger using ctx.stub.putState. After these updates, the corresponding bill is returned in output with JSON format. On the other hand, if the statutes are not ‘verified’ or ‘delivered’, the algorithm throws an exception that only the shipping companies can update the shipping information. This restriction ensures that the shipment details are modified in the appropriate stage of the logistic process.

## 5 System testing and results

To deal with the issues related to transparency, we propose permissioned blockchain networks using Hyperledger Fabric. Our proposed framework has several key properties according to the specific needs of the industry. This framework includes a selected group of participants, including oil carriers at the source, oil refineries, oil distribution terminals, port authorities, customs, oil suppliers, and shipping companies [32]. This curated group ensures that only relevant stakeholders are involved in the network. Moreover, only registered participants can engage in collaborative activities within the blockchain. This ensures a level of trust and accountability among the involved parties. Within this network, specific roles and responsibilities are allocated to different participants. For instance, only port authorities can create new information or bills of lading, while oil suppliers are tasked with updating product information. Similarly, customs departments can only update customs-related information, and shipping companies are responsible for creating shipping-related information. Despite these specific roles, all participants can view transactional data within the network [33].

### 5.1 Simulation setup

To evaluate the proposed framework, we deploy distinct smart contracts. Through these smart contracts, stakeholders execute various tasks. To ensure authorized participation, we issue valid certificates. Unauthorized users are denied access by the smart contract, generating an error message. Moreover, we record historical transactions for traceability. Furthermore, the smart contracts encompass multiple conditions for creating and updating bills of lading. The proposed system is tested on Ubuntu 20.0 with 16GB of RAM. The blockchain network comprises four peer nodes, four ordered nodes, and one certificate authority. Additionally, we utilize the CouchDB database for the ledger storage. Within this network architecture, the bill of lading serves as our chaincode. Table 2 provides the details of the simulation parameters and blockchain network.

**Table 2:** Details of simulation setup and blockchain network.

Parameter	Details	Parameter	Details
Smart Contract Deployment	Distinct smart contracts for various tasks	Blockchain Network Nodes	Four peer nodes, four ordered nodes
Authorized Participation	Valid certificates issued for authorized users	Certificate Authority	One certificate authority
Unauthorized Access	Denied by a smart contract with an error message	Ledger Storage	Utilization of CouchDB database
Transaction Traceability	Historical transactions recorded for traceability	Chaincode	Bill of lading serves as chaincode
Contract Conditions	Multiple conditions for bill of lading operations	System Testing Environment	Ubuntu 20.0 with 16GB of RAM

### 5.2 Resource utilization

**Fig 5:**
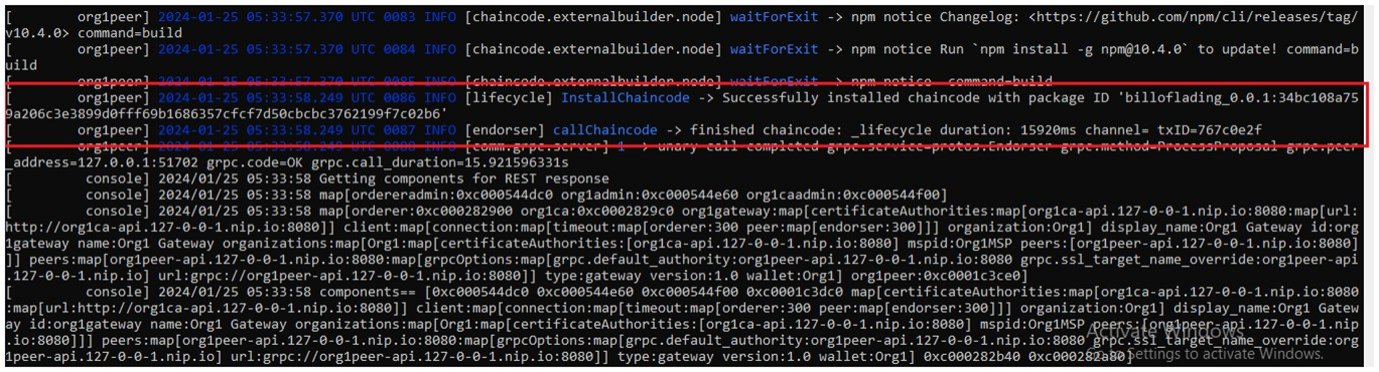
Installation of chaincode on the peer node.

**Fig 6:**
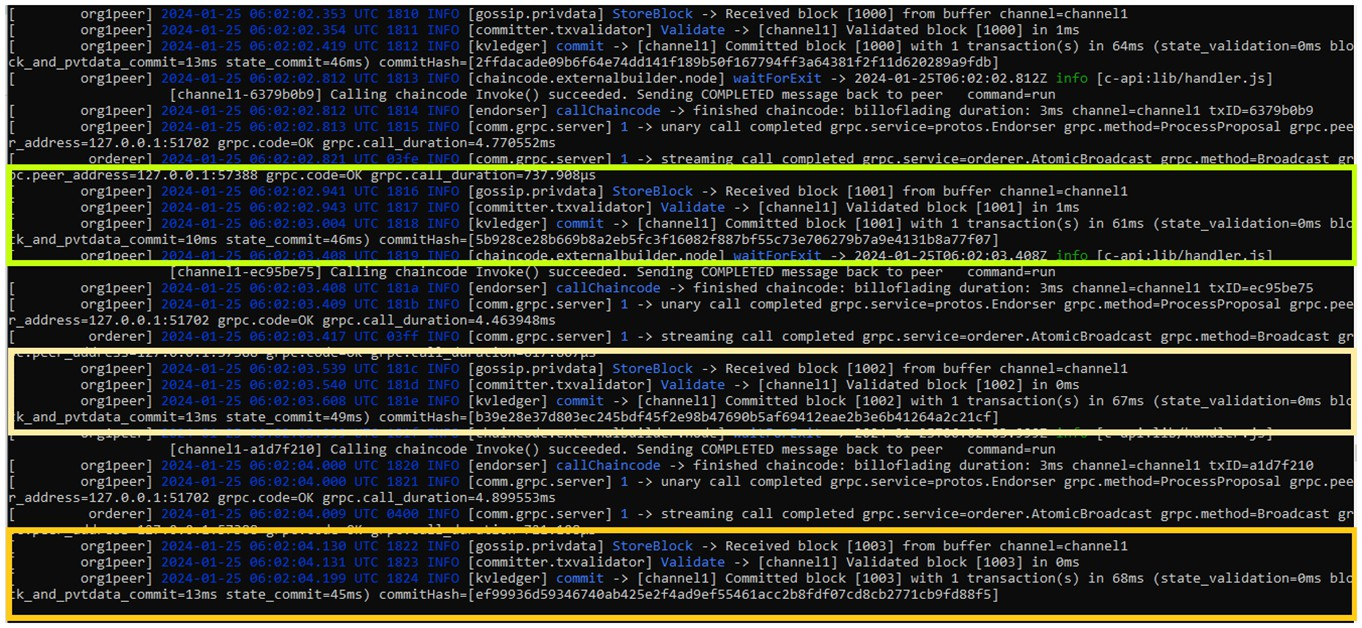
The creation of last three bills.

Fig 5 illustrates chaincode installation on a peer node. This setup ensures the robustness and efficiency of our system for handling transactions and maintaining the integrity of the ledger. Fig 6 illustrates the creation of the last three bills, initiated by the port authorities, i.e., the oil carriers at the source. To validate the efficiency of the proposed work in terms of network latency, throughput, and resource utilization, we tested our system with 1000 transactions to ensure its robustness. The parameters including resource consumption during these transactions are detailed in Table 3, providing valuable insights into the system’s efficiency, speed, and resource utilization. Latency metrics measure the system’s responsiveness in processing transactions, while memory usage metrics offer a comprehensive view of resource utilization throughout the process.

**Table 3:** Resource metrics of the proposed framework.

	Total Time (1000 Transactions)	Maximum Latency	Maximum Memory Usage	Maximum CPU Usage	Throughput
**Transaction Creation**	618105 ms	617909 ms	2363392 bytes	18.70%	1.62 /sec
**Transaction Updation**	603647 ms	603411 ms	10772480 bytes	23.50%	1.66 /sec
**Transaction Verification**	597047 ms	596789 ms	14925824 bytes	40.60%	1.67 /sec

### 5.3 Security analysis

**Fig 7:**
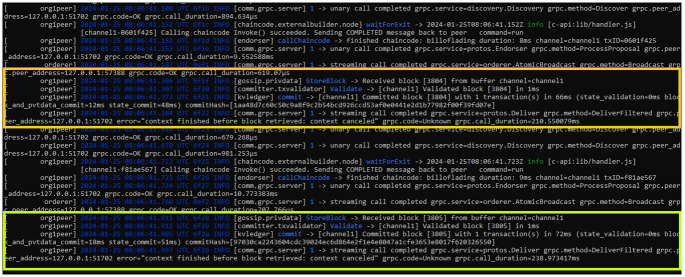
Verification and Validation process of bills by the authorized entities.

We ensure that the data stored on the blockchain remains secure by encrypting it, thus making it impossible to alter. Additionally, we store data in the form of hashes, employing the SHA 256 algorithm. To ensure trustworthiness, we restrict transaction privileges to authorized entities. Fig 7 illustrates the verification and validation process of bills by authorized entities. As mentioned earlier, only authorized entities can initiate and validate the bills at their respective stages and requirements. The oil refinery, upon request initiated by the oil carrier at the source, verifies the bills. Similarly, the oil distribution terminal verifies the bill and grants access upon the carrier’s request. Subsequently, the oil carrier at the destination requests access to the bill, and upon obtaining access, performs the final verification and initiates the shipment delivery. Additionally, customs and other relevant authorities may also verify these bills according to their requirements. Furthermore, Table 3 provides insights into the resource metrics during this verification and validation process. Here it is worth noting that resource consumption is almost similar to transaction creation due to the comparable computational demands of verifying and updating bill information across the distributed ledger. In simple, the proposed systems maintain consistent efficiency throughout various stages of bill handling and validation.

**Fig 8:**
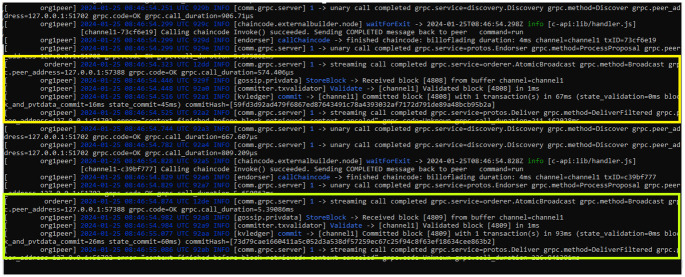
Updating process of bills by the authorized entities.

Fig 8 shows the bill updating process by the respective entities. In our implementation, we ensure that only stakeholders have the authority to update bill information. We also ensure that the entities are restricted to updating parameters related to their specific concerns. For example, we restrict that shippers can only modify shipment information, while customs departments can update customs and tax parameters. Our approach ensures data integrity and accuracy by allowing stakeholders to focus on the specific aspects of the bill that are associated with their needs and roles. Another example is that the shippers can update only the shipment details. This ensures that the information reflects the actual status of the shipment. Similarly, we enable customs departments to modify customs and tax parameters to ensure compliance with regulatory requirements and to facilitate smooth transactions. In simple, our approach enhances operational efficiency and ensures that each entity’s modification is accurately reflected in the bill’s information. Lastly, we also provide the resource statistics and consumption of bill updating by various entities in Table 3.

## 6 Conclusion

We proposed a framework with blockchain technology to address the complexities and vulnerabilities in trade document processes of oil port logistics to overcome the traditional paper-based documentation system as these systems posed significant challenges, including slowness, errors, and susceptibility to manipulation. By implementing a private blockchain, our framework facilitated the automation of smart contracts, ensuring that all stakeholders involved in the logistics process adhered to predefined rules and regulations. Our framework not only streamlined the execution of trade documents but also ensured trust among the involved entities through data immutability. Additionally, smart contracts automated payment processes, mitigating the risks associated with disputes and delays. In the future, we aim to deploy the proposed framework in other applications of oil port logistics such as leakage detection in gas pipelines, managing oil and gas asset lifecycle, traceability of well abandonment and restoration, collection and disposal of wastes at oil refinery stations, and optimization of supply chain logistics.
